# Spinal β-adrenergic receptors’ activation increases the blood glucose level in mice

**DOI:** 10.1080/19768354.2017.1345788

**Published:** 2017-07-10

**Authors:** Yun-Beom Sim, Soo-Hyun Park, Sung-Su Kim, Su-Min Lim, Jun-Sub Jung, Naveen Sharma, Hong-Won Suh

**Affiliations:** Department of Pharmacology, Institute of Natural Medicine, College of Medicine, Hallym University, Chuncheon, Republic of Korea

**Keywords:** Spinal cord, intrathecal, blood glucose, β-adrenergic receptors, pertussis toxin

## Abstract

We examined the role of spinally located β-adrenergic receptors in the regulation of the blood glucose level. The intrathecal (i.t.) injections with dobutamine (β_1_-adrenergic receptor agonist) or terbutaline (β_2_-adrenergic receptor agonist) caused an elevation of the blood glucose level, whereas metoprolol (β_1_-adrenergic receptor antagonist) or butoxamine (β_2_-adrenergic receptor antagonist) did not. In addition, i.t. pretreatment with pertussis toxin (PTX) attenuated the hyperglycemic effect induced by dobutamine or terbutaline. Moreover, plasma insulin level was increased by dobutamine but not by terbutaline, and PTX reduced dobutamine-induced up-regulation of the plasma insulin level. Terbutaline significantly increased plasma corticosterone level, and PTX further enhanced terbutaline-induced corticosterone level. Furthermore, intraperitoneal (i.p.) pretreatment with hexamethonium- (a preganglionic blocker) attenuated dobutamine- and terbutaline-induced hyperglycemic effects. Our results suggest that activation of spinal β_1_- and β_2_-adrenergic receptors produces hyperglycemic effects in a different manner. Spinally located PTX-sensitive G-proteins appear to be involved in hyperglycemic effect induced by terbutaline. Furthermore, dobutamine- or terbutaline-induced hyperglycemia appears to be mediated through the spinal nerves.

## 1. Introduction

Norepinephrine or epinephrine exerts a variety of physiological functions and the activation of adrenergic receptors by adrenergic receptor agonists produces a several pharmacological effects (Aston-Jones et al. 1999; Ramos and Arnsten 2007; Hysek et al. 2012). Epinephrine binds to the β-adrenergic receptor of the pancreatic α-cell (Schuit and Pipeleers 1986), leading to protein kinase A (PKA)-dependent enhancement which causes the increase of cAMP and exocytotic response (Gromada et al. 2001). Several lines of evidence suggest that β-adrenergic receptor appears to be important for the regulation of the glucose level and metabolism. For example, the elevation of insulin and the reduction of blood glucose levels are observed in β-adrenergic receptor knockout mouse (Asensio et al. 2005). In addition, isoprenaline (a β-adrenergic receptors agonist) shows hyperglycemia and provokes a considerable elevation of insulin, and propranolol (a β-adrenergic receptor antagonist) partially and temporarily inhibits the secretion of insulin (Loubatieres et al. 1971; John et al. 1990). Furthermore, ritodrine (a β_2_-adrenergic receptor agonist) decreases glucose concentration and increases blood insulin level under fasting condition (Tsuchiya et al. 2012). A recent study has reported that β-adrenergic receptors are up-regulated in the skeletal muscle in the streptozotocin-induced diabetic model (Xavier et al. 2012).

In addition to the involvement of β-adrenergic systems in the peripheral system, several lines of evidence have suggested that the β-adrenergic system located in the brain also appears to be involved in the regulation of blood glucose level. For example, intracranial isoproterenol produces hyperglycemia (Gunion et al. 1992). In addition, Fóscolo et al. have demonstrated that brain is an important site for the regulation of blood glucose level (Foscolo et al. 2003). They have observed that the administration of norepinephrine or isoproterenol (β-adrenergic receptor agonist) into the medial preoptic area causes an elevation of the blood glucose level, although they shows differences in both magnitude of the blood glucose increase and duration of action. Furthermore, pancreatectomy causes the alterations of β-adrenergic receptor numbers in the cerebral cortex as well as in the hypothalamus (Das et al. 2006).

Although the involvement of peripherally located β-adrenergic receptors as well as β-adrenergic receptors located in the brain sites in the regulation of the blood glucose level has been well demonstrated in numerous previous studies, the possible roles of β-adrenergic receptors located in the spinal cord for the regulation of the blood glucose level have not been well characterized. Thus, in the present study, effects of β-adrenergic receptor agonists and antagonists administered spinally on the blood glucose level were examined in mice.

## 2. Material and methods

These experiments were approved by Hallym University Animal Care and Use Committee (Registration Number: Hallym 2009–05–01). All procedures were conducted in accordance with ‘Guide for Care and Use of Laboratory Animals’ published by National Institutes of Health.

### 2.1. Experimental animals

Male Hsd: CD-1 (ICR) [Charles River, USA] mice, weighing 24–26 g, were used for all the experiments. Five mice were housed per cage in a room maintained at 22 ± 0.5°C with an alternating 12 h light–dark cycle. Food and water were available ad libitum. The animals were allowed to adapt to the laboratory for at least 2 h before testing and were only used once. Experiments were performed during the light phase of the cycle (10:00–17:00). All used mice were sacrificed by CO_2_ inhalation.

### 2.2. Drugs

Dobutamine (selective β-adrenergic receptor agonist; cardiotonic agent), terbutaline (selective β_2_-adrenergic receptor agonist; used as a fast-acting bronchodilator or short-term asthma treatment), metoprolol (selective β_1_-adrenergic receptor antagonist; treat high-blood pressure, chest pain due to poor blood flow to the heart) and butoxamine (selective β_2_-adrenergic receptor antagonist; not clinically) were purchased from Sigma Co. (St. Louis, MO, USA). Pertussis toxin (PTX; a protein-based AB_5_-type exotoxin) was purchased from Tocris Co. (Minneapolis, MN, USA). Hexamethonium (non-depolarising ganglionic blocker) was purchased from Santa Cruz (Ave. Delaware, CA, USA). Dobutamine, terbutaline, metoprolol, butoxamine, hexamethonium and PTX were dissolved in saline. All drugs were prepared just before use. A blood glucose meter, a lancing device and strips were purchased from Roche Diagnostics (Sandhofer Strasse, Mannheim, Germany). The mouse insulin ELISA kit was purchased from Shibayagi Co. (Shibukawa, Japan).

### 2.3. Intraperitoneal (i.p.) and intrathecal (i.t.) injections

I.p. injection was conducted to unanesthesized mice with a volume of 250 µl. I.t. administration was performed in conscious mice, following the method of Hylden and Wilcox, using a 30-gauge stainless-steel needle attached to a 25 µl Hamilton microsyringe (Hylden and Wilcox 1981). The mice were gently handled and i.t. injection was performed as quickly as possible so that the mice can avoid the stress as much as possible. The i.t. injection volume was 5 µl and the injection site was verified by injecting a similar volume of 1% methylene blue solution and determining the distribution of the injected dye in the spinal cord. The i.t. injected dye was distributed both rostrally and caudally but within a short distance (about 0.5 cm), and no dye was found in the brain. The success rate of injections was consistently found to be over 95% before performing the experiments.

### 2.4. Treatment of drugs

In the first experiment, each mouse was administered i.t. with β-adrenergic receptor agonists or antagonists. In the second experiment, mice were pretreated i.t. once with PTX (0.05 or 0.1 µg/5 µl) for 6 days before i.t. administration of vehicle as a control or a fixed dose of dobutamine (5 µg/5 µl) or terbutaline (10 µg/5 µl). In the last experiment, mice were pretreated i.p. with either saline or hexamethonium (1–20 mg/kg) for 20 min before i.t. administration with dobutamine or terbutaline. In addition, every experiment contained saline control made up in physiologic saline (0. 9% NaCl).

### 2.5. Measurement of blood glucose level

Blood glucose measurements were obtained using blood samples collected by lateral tail vein laceration. A minimum volume (1 µl) of blood was collected as quickly as possible. Glucose level was measured using Accu-Chek Performa blood glucose monitoring system (Sandhofer Strasse, Mannheim, Germany).

### 2.6. Insulin ELISA assay

Plasma Insulin level was measured 30 min after i.t. injection with dobutamine or terbutaline. Four hundred microliters of blood was collected by puncturing the retro-orbital venous plexus. Plasma was separated by centrifugation and stored at −80°C until assayed. Measurement of serum level of insulin was performed according to the manufacturer’s manual. The level of insulin in the serum was evaluated by measuring the absorbance at 450 nm using a microplate spectrophotometer Epoch (Biotek, Winooski, VT).

### 2.7. Corticosterone assay

Plasma corticosterone level was measured 30 min after i.t. injection with dobutamine or terbutaline. Four hundred microliters of blood was collected by puncturing the retro-orbital venous plexus. Plasma was separated by centrifugation and stored at −80°C until assayed. Plasma corticosterone levels were measured by using sulfuric acid, and determined by measuring the absorbance at EX475 nm and EM530 nm the fluorometric determination method (Glick et al. 1964).

### 2.8. Statistical analysis

Statistical analysis was carried out by Anova (Boneferroni test) for multiple comparisons, by using GraphPad Prism Version 4.0 for Windows (GraphPad Software, San Diego, CA, USA). *P*-values of less than .05 were considered to indicate statistical significance. All values were expressed as the mean ± S.E.M. In our study, we established the mean blood glucose value of the control group through many experiments under matching condition. Selected mice of the established blood glucose level were then used in replication experiments.

## 3. Results

### 3.1. Effect of β_1_- and β_2_-adrenergic receptor agonist or antagonist administered i.t. on the blood glucose level.

**Figure 1.**
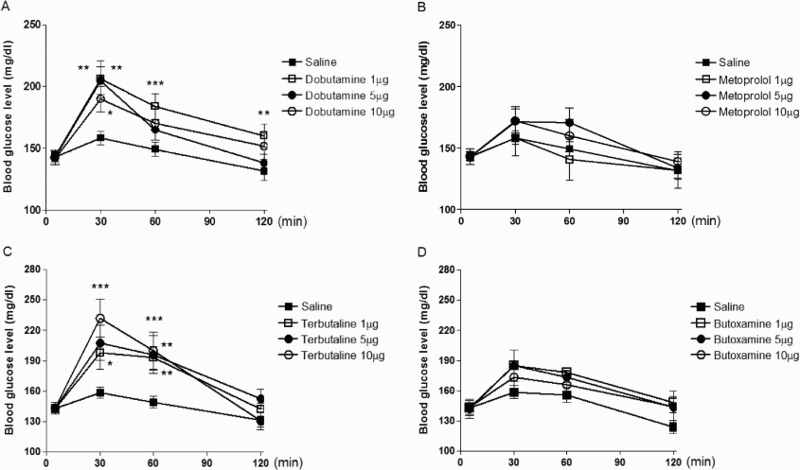
Effects of β_1_- and β_2_-adrenergic receptor agonists and antagonists administered i.t. on the blood glucose level. Mice were treated i.t. with dobutamine (1–10 µg/5 µl) (A) metoprolol (1–10 µg/5 µl) (B), terbutaline (1–10 µg/5 µl) (C), or butoxamine (1–0 µg/5 µl) (D). The blood glucose level was measured at 30, 60 and 120 min after drug injection. The blood was collected from the tail-vein. The vertical bars indicate the standard error of mean. (*P < .05, **P < .01, ***P < .005; compared to saline group). The number of animals used in each group was 8–10.

To investigate the role of spinal β-adrenergic receptors in the regulation of the blood glucose level, mice were treated i.t. with various doses of dobutamine (1–10 µg/5 µl) or metoprolol (1–10 µg/5 µl) and terbutaline(1–10 µg/5 µl) or butoxamine (1–10 µg/5 µl). As shown in Figure 1(A,C), the blood glucose level was significantly increased by dobutamine or terbutaline. Dobutamine- and terbutaline-induced blood glucose level reached at maximal level at 30 min and returned almost to the control level at 120 min after drug injection, In contrast to the result with β-adrenergic receptor agonists, i.t. administration with metoprolol or butoxamine alone did not have any effect on the blood glucose level (Figure 1(B,D)).

### 3.2. Effect of i.t. pretreatment with PTX on increased blood glucose level induced by β-adrenergic receptor agonists administered i.t.

**Figure 2.**
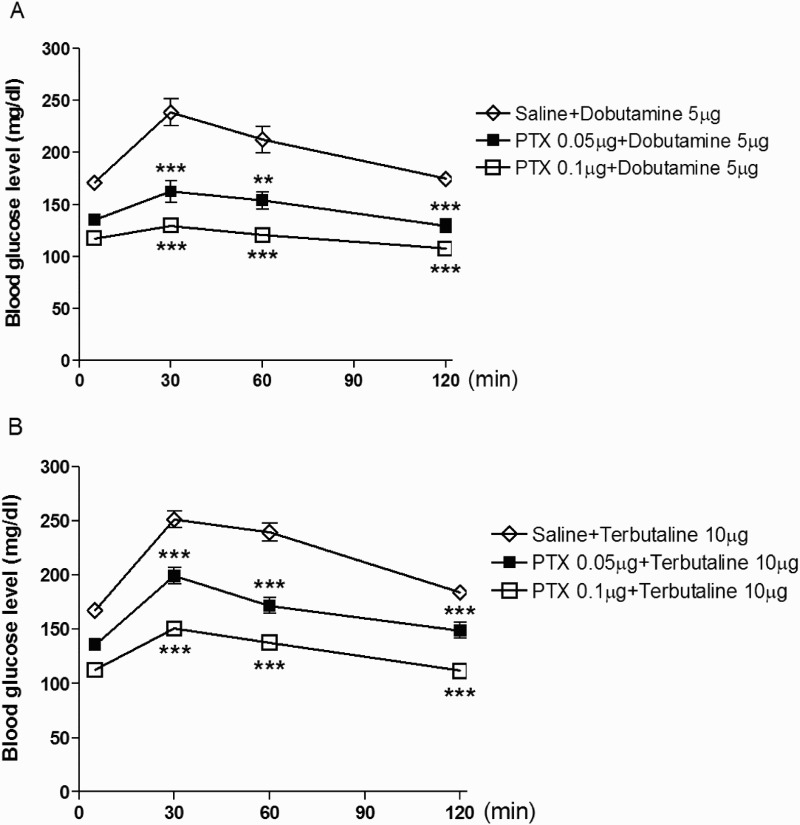
Effect of i.t. pretreatment with PTX on i.t. administered dobutamine or terbutaline-induced blood glucose level. PTX was pretreated i.t. with 0.05 or 0.1 µg/5 µl) for 6 days and then, dobutamine (5 µg/5 µl) (A) or terbutaline (10 µg/5 µl) (B) was administered i.t. The blood glucose level was measured at 30, 60 and 120 min after dobutamine or terbutaline injection. The blood was collected from the tail-vein. The vertical bars indicate the standard error of mean. ((A) **P < .01, ***P < .005; compared to saline + dobutamine group, (B) ***P < .005; compared to saline + terbutaline group). The number of animals used in each group was 8–10.

To investigate the possible role of spinal PTX-sensitive G-proteins in the regulation of the blood glucose in β-adrenergic receptor agonists-induced hyperglycemia, PTX (0.05 or 0.1 µg/5 µl) was pretreated i.t. once for 6 days and then, dobutamine (5 µg/5 µl) or terbutaline (10 µg/5 µl) was administered i.t. As shown in Figure 2(A,B), the i.t. pretreatment with PTX at the dose of 0.05 or 0.1 µg significantly attenuated the elevation of the blood glucose level induced by dobutamine or terbutaline.

### 3.3. Effect of i.t. pretreatment with PTX on plasma insulin and corticosterone levels induced by β-adrenergic receptor agonists administered i.t.

**Figure 3.**
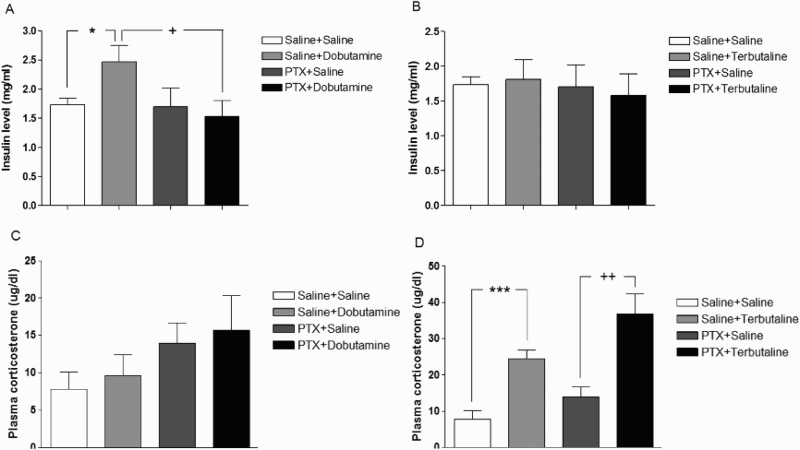
Effect of PTX pretreated i.t. on dobutamine or terbutaline-induced plasma insulin and corticosterone levels. PTX was pretreated i.t. with 0.1 µg/5 µl for 6 days and then, dobutamine (5 µg/5 µl) (A, C) or terbutaline (10 µg/5 µl) (B, D) was administered i.t. The plasma insulin (A, B) and corticosterone (C, D) levels were measured at 30 min after dobutamine or terbutaline injection. The vertical bars indicate the standard error of mean. ((A) *P < .05; compared to saline + saline vs saline + dobutamine group, +*P* < .05; compared to saline + dobutamine vs PTX+dobutamine group; (D) ***P < .005; compared to saline + saline vs saline + terbutaline group, ++*P* < .01; compared to PTX + saline vs PTX + terbutaline group). The number of animals used in each group was 8–10.

To investigate the role of spinal inhibitory G-proteins in β-adrenergic receptor agonists-induced hyperglycemia, the mice were pretreated i.t. with PTX (0.1 µg/5 µl) for 6 days, dobutamine (5 µg/5 µl) or terbutaline (10 µg/5 µl) was administered i.t. As shown in Figure 3(A), the plasma insulin level was significantly increased by dobutamine. The i.t. treatment with PTX alone did not affect the plasma insulin level. However, i.t. pretreatment with PTX reduced the up-regulation of the plasma insulin level induced by dobutamine administered i.t. In contrast, i.t. injection with terbutaline did not have any effect on the plasma insulin level (Figure 3(B)). To examine if the glucocorticoid system is involved in β-adrenergic receptor agonists-induced hyperglycemia, effect of dobutamine and terbutaline administered i.t. on plasma corticosterone level was observed. As shown in Figure 3(C,D), i.t. administration with terbutaline, but not dobutamine, increased plasma corticosterone level, and pretreatment with PTX further enhanced terbutaline-induced up-regulation of plasma corticosterone level.

### 3.4. Effect of hexamethonium pretreated i.p. on hyperglycemic effect induced by β-adrenergic receptor agonists administered i.t.

**Figure 4.**
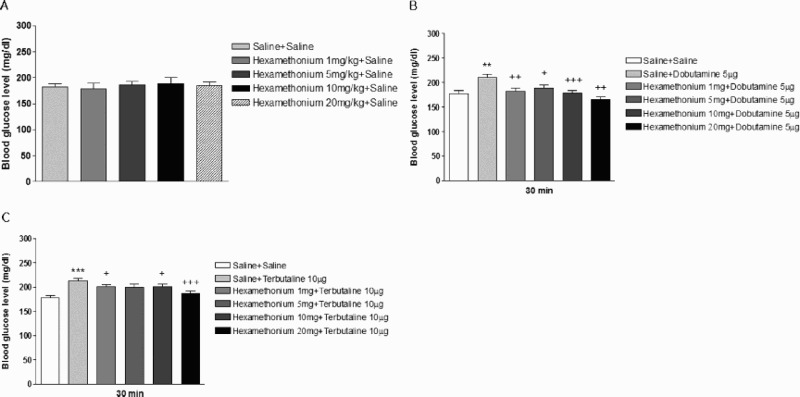
Effect of i.p, pretreatment with hexamethonium on i.t. administered dobutamine- or terbutaline-induced blood glucose level. Hexamethonium (1–20 mg/kg) (A) was pretreated i.p. for 20 min and then, dobutamine (5 µg/5 µl) (B) or terbutaline (10 µg/5 µl) (C) was administered i.t. The blood glucose level was measured at 30 min after dobutamine or terbutaline injection. The blood was collected from the tail-vein. The vertical bars indicate the standard error of mean. ((B) **P < .01; compared to saline + saline group, +*P* < .05, ++*P* < .01, +++*P* < .005; compared to saline + dobutamine group, (C) ***P < .005; compared to saline + saline group, +*P* < .05, +++*P* < .005; compared to saline + terbutaline group). The number of animals used in each group was 8–10.

Hexamethonium is a ganglionic blocker which does not cross the blood brain barrier. To examine if peripheral spinal nerves are involved in the production of hyperglycemia induced by β-adrenergic receptor agonists administered spinally, mice were pretreated i.p. with various doses of hexamethonium (1 to 20 mg/kg) and then, dobutamine (5 µg/5 µl) or terbutaline (10 µg/5 µl) was administered i.t. As shown in Figure 4(A), the i.p. treatment with hexamethonum alone did not affect the blood glucose level. However, the i.p. pretreatment with hexamethonium attenuated the hyperglycemic effect induced by dobutamine or terbutaline administered i.t. as revealed in Figure 4(B,C).

## 4. Discussion

Previous studies have demonstrated that the blood glucose level is regulated by β-adrenergic receptor agonists or antagonists administered systemically or supraspinally (Ferlito et al. 1985; Foscolo et al. 2003). The physiological significance of β_1_- or β_2_-receptors has used clinical medicine. Several selective antagonists for the β_1_-adrenergic receptor are at the front medication for chronic heart failure, coronary heart disease or hypertension patients, on the other hand β_2_-adrenergic receptor agonists play an important role for asthma therapy (Frishman and Lazar 1990; Psaty et al. 1997; Brophy et al. 2001). Our present and previous studies suggest that the spinal cord plays an important role for the regulation of the blood glucose level. We have recently reported that IL-1β administered spinally causes an elevation of the blood glucose level (Sim et al. 2012a). In addition, spinal administration of anti-diabetic agents like biguanide, thizolidinediones or repaglidine modulates the blood glucose level, whereas ghrelin receptors located in the spinal cord plays important roles in the elevation of the blood glucose level (Sim et al. 2012b; Sim et al. 2013; Sim et al. 2014a). In an attempt to characterize the role of the spinally located β-adrenergic receptors in the regulation of blood glucose and insulin levels, the effects of β-adrenergic receptor agonists or antagonists administered spinally on blood glucose and insulin levels were examined in the present study. We found for the first time that spinal treatment with dobutamine or terbutaline increases blood glucose level. However, metoprolol or butoxamine administered i.t. does not affect blood glucose level. Our results are in part in line with a previous study by Foscolo et al. (2003). They have reported that systemic injection of isoproterenol, β-adrenergic agonist, only produces a small, transient increase in plasma glucose level. Thus, it can be speculated that the hyperglycemic effect induced by isoproterenol administered systemically might be, in part, mediated by an activation of β_1_- or β_2_-adrenergic receptors located in the spinal cord. Detailed study to delineate this hypothesis should be further performed in future study. The finding of the lack of glycemic effect of metoprolol and butoxamine suggests that β_1_- or β_2_-adrenergic receptors in the spinal cord are not tonically involved in the regulation of blood glucose level.

Several lines of evidence have demonstrated that PTX-sensitive G-proteins are involved in blood glucose regulation. For example, PTX administered systemically produces hypoglycemic effect in an *in vivo* study (Toyota et al. 1980; Garcia Hermida et al. 1991). Furthermore, in an *in vitro* study, PTX appears to increase the secretion of insulin (Komatsu et al. 1995). To investigate the possible role of spinal PTX-sensitive G-proteins in β-adrenergic receptor agonists-induced hyperglycemic effect, PTX was pretreated spinally and its action on β-adrenergic receptor agonists-induced hyperglycemia was assessed. The spinal pretreatment with PTX alone produces hypoglycemia, which has been revealed though earlier studies (Sidney et al. 1987; Sim et al. 2014b). Spinally pretreated PTX at a lower dose (0.05 µg) slightly attenuated a hyperglycemic effect induced by dobutamine or terbutaline, whereas both dobutamine and terbutaline could not increase blood glucose level at a higher dose (0.1 µg). In addition, spinal treatment with dobutamine increases the plasma insulin level, and spinal pretreatment with PTX reduced up-regulation of the plasma insulin level induced by dobutamine injected spinally, whereas spinal administration with terbutaline did not affect the plasma insulin level. This finding led us to suggest that increased insulin level by dobutamine may be due to the production of hyperglycemia induced by dobutamine. Furthermore, the reduction of dobutamine-induced insulin level by PTX appears to be due to inhibiting effect of PTX-against dobutamine-induced hyperglycemia.

Moreover, the glucocorticoid system has been implicated for the modulation of blood glucose level in diabetes. Plasma corticosterone level is significantly enhanced in streptozotocin-administrated rats or diabetic mice (Coleman and Burkart 1977; Schwartz et al. 1997). We found in the present study that spinal administration with terbutaline significantly increases the plasma corticosterone level, whereas spinal administration with dobutamine does not affect plasma corticosterone level. Thus, it is suggested that increased corticosterone level may lead to a terbutaline-induced hyperglycemia, whereas the corticosterone system is not involved in the regulation of dobutamine-induced hyperglycemia. In addition, we found that PTX pretreatment causes further enhancement of corticosterone level induced by terbutaline, but not dobutamine. Thus, it is speculated that an enhancing effect of PTX on terbutaline-induced corticosterone level may be attributed to a negative feedback mechanism involved in the inhibiting action of PTX against terbutaline-induced hyperglycemic effect. This finding suggests that the glucocorticoid system may actively participate in regulation of β_2_-adrenergic receptor agonist-induced hyperglycemia.

Taken together, our results suggest that, although both dobutamine and terbutaline cause hyperglycemia, differential mechanisms appear to be involved in their pharmacological actions. In addition, although PTX pretreatment attenuates hyperglycemia effects of both dobutamine and terbutaline, PTX may inhibit their hyperglycemic effects in a different manner. Since it has been well known that activation of β_2_-adrenergic receptors is linked with Gi protein (Liu et al. 2009), PTX-sensitive G-proteins located at the spinal cord appear to be involved in the production of hyperglycemia induced by the activation of β_2_-adrenergic receptors by terbutaline. However, the exact mechanism involved in PTX-induced antagonism against dubutamine-induced hyperglycemic effect should be further characterized.

Hexamethonium is known as a preganglionic blocker and cannot cross the blood brain barrier (Malin et al. 1997). There exist both β_1_- and β_2_-adrenergic receptors in the spinal cord (Schrader and Grobecker 1989) and their functions associated with spinal nerves have been previously reported (Brown & Dunn 1983; Linderoth et al. 1994). To examine if the hyperglycemic effect induced by dobutamine or terbutaline administered spinally is mediated through the peripheral spinal nerves and preganglions, we challenged various doses of hexamethonium systemically and examined its effect on hyperglycemia induced by dobutamine or terbutaline. The i.p. treatment with hexamethonium alone did not have any effect on the blood glucose level. Additionally, we found in the present study that systemic pretreatment with hexamethonium significantly attenuates the hyperglycemic effect induced by dobutamine or terbutaline administered spinally. These findings suggest that peripheral spinal ganglions appear to be involved in spinally administered dobutamine- or terbutaline-induced hyperglycemic effect.

In conclusion, our results suggest that β_1_- and β_2_-adrenergic receptors located in the spinal cord play important roles in the elevation of blood glucose level in a different manner. Spinally located PTX-sensitive G-proteins appear to be involved in hyperglycemic effect induced by terbutaline administered spinally. Furthermore, spinal nerves appear to be involved in spinally given dobutamine- or terbutaline-induced hyperglycemia.
